# Genetic and maternal predictors of cognitive and behavioral trajectories in females with fragile X syndrome

**DOI:** 10.1186/s11689-018-9240-2

**Published:** 2018-06-20

**Authors:** Laura del Hoyo Soriano, Angela John Thurman, Danielle Jenine Harvey, W. Ted Brown, Leonard Abbeduto

**Affiliations:** 1 MIND Institute, University of California Davis, 2825 50th Street, Sacramento, CA 95817 USA; 2 Department of Psychiatry and Behavioral Sciences, University of California, Davis, Sacramento, USA; 3 Division of Biostatistics, Department of Public Health Sciences, University of California, Davis, CA USA; 4 NY Institute for Basic Research on Developmental Disabilities, Staten Island, NY USA

**Keywords:** Females with FXS, Ratio of affected to total chromosomes, FMRP, Maternal psychological distress, Closeness in the mother–child relationship, Fluid intelligence, Crystallized intelligence, Anxiety, Withdrawal, Longitudinal

## Abstract

**Background:**

Fragile X syndrome (FXS) is caused by a mutation in the *FMR1* gene on the X chromosome, leading to decreased levels of FMR1 protein (FMRP), which causes the array of neuropsychological impairments that define FXS. Because FXS is an X-linked condition, fewer females display FXS and females with FXS are more mildly affected than males, on average. However, there is a considerable variability in terms of severity of affectedness among females with FXS. The current study was designed to investigate potential genetic (FMRP level and *ratio of affected to total chromosomes*) and environmental factors (maternal psychological distress and closeness in the mother–child relationship) influencing the cognitive (fluid and crystallized intelligence) and behavioral (anxiety and withdrawal) phenotype of females with FXS.

**Methods:**

We conducted a prospective 3-year longitudinal study of 16 females with FXS (with up to four assessments, each separated by a year) using an accelerated longitudinal design so that we had coverage of the age range of 10–15 years at study start and 13–18 at study end. We focused on both the level of functioning related to chronological age expectations (standard scores) and absolute change in skill (raw scores) over the 3-year period.

**Results:**

At a cross-sectional level, fluid intelligence and crystallized intelligence were both predicted by a closer mother–child relationship and lower maternal psychological distress. However, only fluid intelligence was predicted by a lower ratio of affected to total chromosomes. Anxiety and withdrawal were predicted by a higher ratio of affected to total chromosomes. Withdrawal was also predicted by lower closeness in the mother–child relationship and higher maternal distress. In terms of longitudinal change, gains were observed in fluid and crystallized intelligence, whereas anxious and withdrawn behaviors remained stable over visits. Gains in fluid intelligence were solely predicted by FXS biomarkers (higher FMRP level and lower ratio of affected to total chromosomes), while gains in crystallized intelligence were not predicted by any of the biological and environmental variables.

**Conclusions:**

Our results show that FXS biomarkers and maternal variables contribute differentially to the cognitive and behavioral features of the adolescent female with FXS. These findings can help in the design of treatment studies aimed at enhancing cognitive and behavioral abilities in the FXS population.

## Background

Fragile X syndrome (FXS) is the most common known cause of inherited intellectual disability, occurring in 1 of every 4000 male births and 1 of every 8000 female births [1, 2]. The syndrome is caused by the expansion of a CGG trinucleotide repeat in the *fragile X mental retardation 1* gene (*FMR1*) on the long arm of the X chromosome. In FXS, expansions greater than 200 CGG repeats lead to hypermethylation and silencing of the *FMR1* gene. In the full mutation, there are decreased levels or the complete absence of the FMR1 protein (FMRP), which is essential for synaptic development and plasticity [3]. Reduced FMRP leads to atypical brain development and the array of neuropsychological impairments that define FXS [4, 5]. Because FXS is an X-linked disorder, there are differences in the phenotypes of affected males and females. In the present study, we focused on females with FXS, who have been relatively less studied than males.

The cognitive phenotype of individuals with FXS is characterized by a generalized intellectual delay in comparison to those who are typically developing (TD), although there is considerable phenotypic variation, especially among females. In addition, individuals with FXS demonstrate a unique pattern of cognitive strengths and limitations [6]. Relative weaknesses have been identified in a range of tasks related to visual–motor coordination, visual memory, visual–spatial reasoning, short-term memory, and executive function [7–10]. In contrast, relative strengths (although not to age-appropriate levels) have been observed in verbal ability, acquired knowledge, long-term memory for verbal information, and simultaneous processing [10–13]. In other words, individuals with FXS show relatively less impairment in those tasks that require accessing long-term information, especially verbal information, acquired through explicit learning (so-called crystallized ability) and more severe impairment in logical novel problem solving, abstract reasoning, and formulation of rules (so-called fluid reasoning) [14, 15]. Although fluid and crystallized intelligence skills are correlated, with crystallized intelligence being the product of the synergistic effects between one’s cultural experience and one’s fluid intelligence, these two cognitive domains have different neural substrates and developmental trajectories [16, 17].

In addition to cognitive impairments, behavioral difficulties have been reported for individuals with FXS. These difficulties include anxiety, social withdrawal behaviors, and poor eye contact, all of which emerge as early as 3–5 years of age [18–20]. Indeed, a significant number of individuals with FXS meet criteria for autism spectrum disorder (ASD), and, although the ASD diagnosis is less prevalent among females, many females are likely to display levels of ASD-like behaviors below the diagnostic threshold. These difficulties, which can be present even in females with IQs in the average range [21], limit their daily functioning [22] and are a source of stress for families [23].

Determining the developmental course of these cognitive and behavioral features and the factors affecting those trajectories is important for determining the timing and types of interventions likely to be beneficial for individuals with FXS. However, findings to date have been inconsistent across studies as regards these trajectories and their determinants, owing in part to differences in the specific cognitive skills and behaviors examined, the tools of assessment used, the context of assessment, the statistical approaches taken, the characteristics of the sample, and the types of scores used [24].

In the case of females with FXS, the presence of a second X chromosome with a healthy *FMR1* gene serves a protective function relative to affected males. Moreover, the process of X inactivation leads to variability among females as regards FMRP expression. In particular, the level of FMRP will depend on the proportion of cells that have the unaffected X chromosome as the active X chromosome. As a consequence, females with FXS show a wider range of variability in FMRP production than males, in addition to higher FMRP concentrations than males on average [5]. This FMRP-related difference is reflected in the phenotype of females with FXS [5, 25, 26]. In particular, previous studies have shown that fluid intelligence [27] and behavioral skills [28] in females with FXS increase linearly as level of FMRP increases. Compared to males with FXS, females with FXS show higher levels of intellectual functioning and lower rates of behavioral problems on average [6]. In females with FXS, for example, fewer than half meet criteria for an intellectual disability compared to males with FXS, of whom virtually all meet criteria for an intellectual disability [29–31].

In addition to genetic/biological correlates of phenotypic variation, environmental factors have been shown to modulate the cognitive and behavioral phenotypes of children with FXS. Developmental outcomes for children with intellectual disability are known to be improved when parents establish a positive family environment. Highly supportive parenting, as well as better maternal mental health, are associated with lower levels of behavioral problems and enhanced verbal skills [32–34] in females and males with FXS.

It is important to recognize that the degree of impairment and the trajectory of development in cognition and behavior may change over the course of the lifespan. Moreover, the contributions of genetic and environmental factors to development might vary across different developmental periods as well. Although it is important to address these relationships in any developmental period, adolescence is of special interest because it is a transition period to a more independent adult life [35]; however, this period has seldom been explored for FXS, especially for females with FXS.

In one of the few studies to focus on females, Kover et al. [27] examined several predictors of the level and trajectory of fluid reasoning in 53 adolescents with FXS (37 males, 16 females) in a longitudinal design. Although the primary analyses focused on the whole sample, separate exploratory analyses were conducted for males and females. These investigators examined the predictors of the level of, and change over time in fluid reasoning using the variables of FMRP, degree of ASD symptom severity, and socioeconomic status. Kover et al. found that FMRP level predicted level of fluid reasoning ability for females with FXS, with no other significant relationships emerging. Kover et al. also examined visualization abilities, but there were no significant findings for this measure for females.

The current study was designed to build on the study by Kover et al. [27] and extend our knowledge of females with FXS by expanding the set of predictors and dependent variables. We focused on a set of genetic and maternal-related environmental predictors and examined their relationship to crystallized intelligence (a non-verbal domain of relative strength), fluid intelligence (a verbal domain of relative weakness), and problems in anxiety and social withdrawal in adolescent females with FXS. We conducted a prospective 3-year longitudinal study (with up to four assessments per participant including baseline and annual visits). We used an accelerated longitudinal design so that we had coverage of the age range of 10–15 years at study start and 13–18 at study end. The goals were to establish the trajectory of cognitive and behavioral development during the adolescent period in females with FXS and to evaluate the contributions of biological and maternal-related environmental variables to the degree of impairment and the trajectory of change in cognition and behavior.

## Methods

### Procedures

Families were recruited for this research through newspaper advertisements, nationwide radio announcements, and a university registry of families with children who have developmental disabilities, as well as through postings on internet sites, listservs, and newsletters of developmental disability organizations. Prior to being enrolled in the study, parents of all participants signed informed consent forms approved by Institutional Review Boards at the University of Wisconsin–Madison and the New York State Institute for Basic Research. Trained examiners (graduate students in communication disorders, education or a related field) completed all testing in a quiet testing room at the University of Wisconsin–Madison. As needed, participants were provided breaks within sessions and between sessions, with the entire protocol at any annual assessment typically being administered over two consecutive days. In general, the same examiner administered all assessments to any given participant at every annual assessment. Additionally, the scoring of all test protocols was checked by two examiners and all data entry was double-checked by two research assistants. The participants and measures reported on this project are a subset of those previously collected from a larger study (R01HD024356), with several previous reports on the study being published (see [27, 36–38]), although none with the specific focus and measures of the present study.

### Participants

Participants were 16 females with FXS who were assessed at an initial visit and at 1-year intervals over the course of 3 years. Females ranged in age from 10.2 to 15.6 years (M = 12; SD = 1.5) at the time of enrollment and from 13.2 to 18.6 years (M = 15; SD = 1.5) at the end of the study. All were previously diagnosed by an appropriate molecular genetic test as having more than 200 CGG repeats in the *FMR1* gene in at least some cells, with documentation of testing provided by the mother at enrollment. During the course of the project, additional analyses were conducted on peripheral blood to confirm the diagnosis and to derive measures of ratio of affected to total chromosomes and FMRP. For all participants, the mother reported that her daughter (a) used speech as the primary means of communication, (b) regularly communicated with three-word or longer phrases, (c) functioned at a kindergarten level or above in most areas, and (d) had no (uncorrected) sensory or physical impairments that would limit performance in this project. All the participants and their mothers were native English speakers. In all cases, all mothers were the biological mother of the participants with FXS and thus, the mothers were necessarily carriers of an expanded *FMR1* gene.

The participants in this study as well as one of the predictors (FMRP level) and one of the dependent variables (fluid intelligence) were the same as in the study by Kover et al. [27]. However, in the current study, we have expanded the set of dependent variables (added a verbal measure of crystallized ability, as well as anxiety and withdrawn behavior measures), and added new predictors (i.e., ratio of affected to total chromosomes maternal psychological distress, and maternal perceived closeness in the mother–child relationship*)* as described in the sections below.

### Predictors of level of ability and rate of change

We considered a set of genetic and environmental potential predictors, each of which was assessed at the initial visit unless otherwise noted.

#### Biological predictors

##### Level of FMRP expression

Blood samples were obtained from participants to measure the level of FMRP expression upon entry into the study. Following methods by Willemsen et al. and Tassone et al. [39], we determined the proportion of cells that expressed the FMRP protein for each participant (using a sample of 400 cells). The average proportion of cells that expressed the protein was 0.48 (SD = 0.05, range = 0.34–0.51). FMRP levels were not available for two participants due to participant or parent refusal to participate in a blood draw (*n* = 1) or because of technical issues (*n* = 1).

##### Ratio of affected to total chromosomes

The ratio of X chromosomes carrying the full mutation, relative to the total number of X chromosomes sampled per participant, was based on the radioactivity of the bands in a Southern blot using a cloned Pst I fragment (StB12.3) as a probe. As is standard practice, the ratio for the full mutation was inferred from the inactivation ratio of the normal allele. In addition, as a technical correction, the intensities of the normal active and inactive bands in the full mutation were adjusted for the efficiency of Southern transfer using the ratio of the inactive: active bands of normal females on the same Southern blot [40]. The average ratio of affected to total chromosomes was 56.47% (SD = 19.9, range = 27–88).

#### Maternal-related environmental predictors

*Maternal psychological distress* was assessed with The Symptom Checklist—90 Revised (SCL-90-R) [41], a 90-item self-report instrument that covers a range of psychological symptom clusters. Mothers subjectively rated each symptom as 0 (no distress), 1 (a little bit distressed), 2 (moderately distressed), 3 (quite a bit of distress), and 4 (extremely distressed). Nine primary symptom dimensions are assessed (*somaticism*, *obsessive–compulsive*, *interpersonal sensitivity*, *depression*, *anxiety*, *hostility*, *phobic anxiety*, *paranoid ideation*, and *psychoticism)*. The derived *T*-score from the general severity index (GSI) was the variable used in the present study as our index of maternal psychological symptoms of distress.

*Closeness in the mother–child relationship* was measured using the Positive Affect Index (PAI) [42]. Five self-report items that reflected the mother’s perception of the child’s reciprocated closeness (i.e., the mother’s perception of how close she believed her child felt toward her; Child-PAI: sum of items 1 to 5) were used from this 10-item self-report scale. Items rated understanding, trust, fairness, respect, and affection in the relationship on a 6-point scale, with higher ratings reflecting a higher quality of the relationship. Possible scores for Child-PAI range from 5 to 30, with higher scores indicative of a more positive mother–child relationship.

### Dependent variables: cognitive and behavioral functioning and trajectories of participants

The following neuropsychological assessments were administered four times (once annually since enrollment and during the next 3 years).

#### Fluid intelligence

We used the fluid reasoning composite score derived from the Sequential Order (SO) and Repeated Patterns (RP) subtests of the Leiter International Performance Scale-Revised (Leiter-R) [43], which is an individually administered standardized test in which instructions are pantomimed by the examiner and no verbal responses are required from the participant. The SO and RP subtests require identification of patterns or rules. In the SO subtest, the participant identifies the item that completes a visually depicted sequence. For RP, the examinee is shown repetitive sequences of items with missing elements and determines how to order the missing elements so as to retain the pattern.

#### Crystallized intelligence

The Expressive Vocabulary Test (EVT) [44] was administered to all participants in order to assess facility with expressive verbal demands dependent on knowledge from the past as a measure of crystallized ability. In the EVT, expressive vocabulary knowledge is elicited using pictures and examiner prompts.

Both, the fluid reasoning index of the Leiter-R and the EVT total score, have been employed successfully across a variety of clinical populations, including FXS girls of similar ages [6, 27, 37]. In order to evaluate level of functioning relative to chronological age expectations, the fluid reasoning composite standard score and the expressive vocabulary standard score derived from age-based norms (each one having a mean of 100 and standard deviation of 15) were used. In order to evaluate the rate of individual change over time, raw scores (i.e., number of correct items from the Leiter-R SO and RP subtests, and from the EVT) were used [38, 45].

Anxiety and withdrawal were assessed through the Child Behavior Checklist, Ages 6-18 (CBCL/6-18) [46]. The CBCL/6-18 uses 118 items to query caregivers about children’s competencies and behavioral/emotional problems. Caregivers rate the child for how true each item is now or within the past 6 months, using 0 = not true (as far as you know), 1 = somewhat or sometimes true, or 2 = very true or often true. In the present study, we used the total *T*-score from the following subscales: *Anxious/Depressed*; and *Withdrawn/Depressed*, given their clinical relevance for girls with FXS shown in previous studies [28].

### Statistical analysis

Repeated measures and random effects models were used to assess change over time in fluid intelligence, crystallized intelligence, and behavioral problems as well as to assess how the biological and maternal-related environmental variables were associated with level (initial visit) and change over time in these variables. The dependent variables used were children’s fluid intelligence (either standard or raw score), children’s crystallized intelligence (either standard or raw score), and CBCL anxiety *T*-score and CBCL withdrawn *T*-score. The independent variables were (1) level of FMRP expression or ratio of affected to total chromosomes as the biological factor and (2) mother’s perception of the child’s reciprocated closeness on the PAI or maternal psychological distress on the SCL-90-R, as the maternal-related environmental factor*.* For each outcome, we first assessed whether there was any estimated average change over time. Independent variables were all standardized by subtracting the mean and dividing by the standard deviation. Each independent variable was included in a model as a single predictor of the absolute level, and the level of change in the outcome. No joint model including multiple independent variables was fit due to the small sample size. Age at the baseline visit was considered as a potential confounder and included in all models as both a predictor of level and change over time. Random intercepts were included in all models to account for between-person variability in overall starting place. When supported by the data, random slopes were also included to account for between-person variability in change. Robust sandwich estimators were used for standard error estimation due to the small sample sizes. All analyses were conducted in SAS version 9.4, with a *p* value less than 0.05 considered statistically significant.

## Results

### Descriptive statistics

Sociodemographic data are presented in Table 1. Descriptive data at all times of assessment are presented in Table 2.

**Table 1:** Demographics characteristic

	*n* (%)
Children race	
Caucasian	13 (81.25%)
African–American	1 (6.25%)
Unknown	2 (12.5%)
Children living with both parents*	
Yes	12 (75%)
No	3 (18.75%)
Number of siblings*	
One sibling	7 (43.75%)
Two siblings	6 (37.5%)
Three siblings	1 (6.25%)
Four siblings	1 (6.25%)
Family income*	
< $10,000	1 (6.25%)
$30–40,000	1 (6.25%)
$40–50,000	4 (25%)
$50–60,000	3 (18.75%)
$60–70,000	1 (6.25%)
$70–80,000	1 (6.25%)
$90–100,000	2 (12.5%)
$100–110,000	1 (6.25%)
$110–120,000	1 (6.25%)
Maternal level of education*	
Graduated high school	8 (50%)
Graduated college	4 (25%)
Achieved an advanced degree	3 (18.75%)
Maternal occupation*	
Not employed	3 (18.75%)
Part-time employment	4 (25%)
Full-time employment	8 (50%)

*1 missing value

**Table 2:** Descriptive statistics at all times of assessment

Visits→	Visit 1	Visit 2	Visit 3	Visit 4
Variables	Mean (SD) [range]	Mean (SD) [range]	Mean (SD) [range]	Mean (SD) [range]
Chronological age	12 (1.5) [10–16]	13 (1.5) [11–17]	14 (1.5) [12–18]	15 (1.5) [13–19]
Biological predictors				
Ratio of affected to total chromosomes	56.47 (19.92) [27–88]			
Level of FMRP expression	0.48 (0.05) [0.34–0.51]			
Maternal predictors				
Maternal psychological distress	51.5 (11.6) [30–65]			
Closeness in the mother–child relationship	23.5 (4.1) [16–28]			
Dependent variables				
Fluid intelligence (SS)	68 (13.6) [48–90]	70.3 (15.5) [48–100]	62.9 (12.6) [48–88]	66.5 (15.2) [48–102]
Fluid intelligence (RS)	33.9 (12.8) [17–55]	38.9 (11.6) [18–58]	37.4 (13.3) [17–61]	39.8 (13.1) [20–61]
Crystallized intelligence (SS)	81.2 (17.6) [51–107]	84 (16.3) [57–112]	81.3 (15.7) [52–100]	82.7 (20.9) [41–109]
Crystallized intelligence (RS)	87.8 (19.8) [53–120]	95.9 (20.3) [64–132]	96.6 (20.2) [62–135]	103.8 (25) [62–148]
Anxious behaviors (TS)	59.53 (10.01) [50–84]	60 (7.7) [50–72]	60.1 (8.2) [50–74]	58.2 (7.2) [50–70]
Withdrawn behaviors (TS)	62.5 (11.6) [50–87]	59.8 (9.4) [50–81]	64.4 (10.7) [50–81]	62 (11.8) [50–85]

*Abbreviations*: *SS* standardized score (mean of 100, standard deviation of 15), *RS* raw score, *TS T*-score (mean of 50, standard deviation of 10)

### Fluid intelligence and crystallized intelligence at baseline

Standard scores for fluid intelligence and for crystallized intelligence at the baseline visit were both linked to the SCL-90-R GSI T score (*β* = − 8.6, SE = 2.2, *p* < 0.001; *β* = − 13.2, SE = 2.7, *p* < 0.001) and Child-PAI score (*β* = 6.7, SE = 2.8, *p* = 0.02; *β* = 8.8, SE = 2.7, *p* = 0.002). In addition, the ratio of affected to total chromosomes was associated with fluid intelligence standard scores (*β* = − 7.9, SE = 2.1, *p* < 0.001). All data are presented in Table 3. Fluid and crystallized intelligence standard scores did not change significantly over time (*β* = − 0.63, SE = 0.63, *p* = 0.32; *β* = 0.48, SE = 0.51, *p* = 0.34).

**Table 3:** Random effects models of biological and maternal-related environmental variables with level (initial visit) and change over time in dependent variables

	Fluid intelligence		Crystallized intelligence		Withdrawal	Anxiety
	Baseline (SS)	Trajectory (RS)	Baseline (SS)	Trajectory (RS)	Baseline (TS)	Baseline (TS)
Ratio of affected to total chromosomes	*β* = − 7.9, SE = 2.1 *p* < 0.001	*β* = − 1.5, SE = 0.6 *p* = 0.02	*β* = − 4.5, SE = 3.6 *p* = 0.22	*β* = 0.7, SE = 0.9 *p* = 0.45	*β* = 6.9, SE = 1.7 *p* < 0.001	*β* = 5.5, SE = 1.9 *p* = 0.006
Level of FMRP expression	*β* = 0.7, SE = 3.0 *p* = 0.8	*β* = 1.2, SE = 0.4 *p* = 0.01	*β* = 5.3, SE = 2.8 *p* = 0.07	*β* = 0.47, SE = 0.27 *p* = 0.09	*β* = 2.3, SE = 1.7 *p* = 0.17	*β* = 0.5, SE = 1.0 *p* = 0.60
Closeness in the mother–child relationship	*β* = 6.7, SE = 2.8 *p* = 0.02	*β* = 0.2, SE = 20.8 *p* = 0.83	*β* = 8.8, SE = 2.7 *p* = 0.002	*β* = 0.73, SE = 0.39 *p* = 0.07	*β* = − 8.1, SE = 1.7 *p* < 0.001	*β* = − 4.0, SE = 2.0 *p* = 0.053
Maternal psychological distress	*β* = − 8.6, SE = 2.2 *p* < 0.001	*β* = − 0.5, SE = 1.2 *p* = 0.7	*β* = − 13.2, SE = 2.7 *p* < 0.001	*β* = − 0.7, SE = 0.6 *p* = 0.26	*β* = 5.2, SE = 2.2 *p* = 0.02	*β* = 2.5, SE = 2.7 *p* = 0.36

*Abbreviations*: *SS* standardized score (mean of 100, standard deviation of 15), *RS* raw score, *TS T*-score (mean of 50, standard deviation of 10), *β* random slope, *SE* standard error estimation, *p p* value

Italicize data represent *p* values < 0.05

### Trajectory of fluid intelligence and crystallized intelligence

**Fig. 1:**
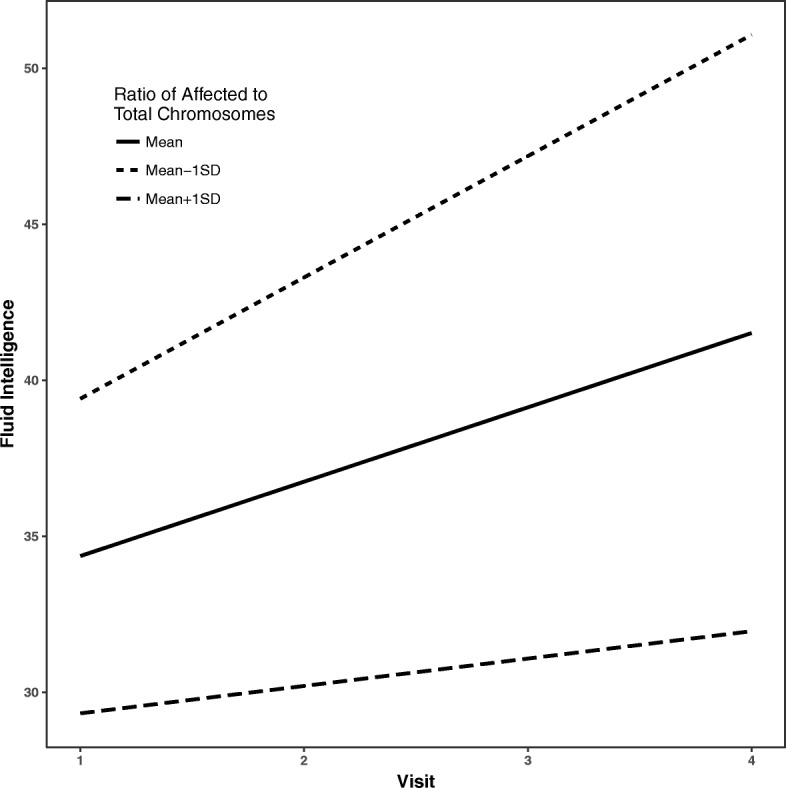
Gains over time in fluid reasoning predicted by X-activation ratio. Legend: estimated average trajectories over time in fluid intelligence for individuals with X-activation ratio at the mean (solid), 1 standard deviation (SD) below the mean (short-dashed) and 1 SD above the mean (long-dashed). Higher ratios of affected to total chromosomes were associated with a slower rate of improvement in fluid intelligence over time

**Fig. 2:**
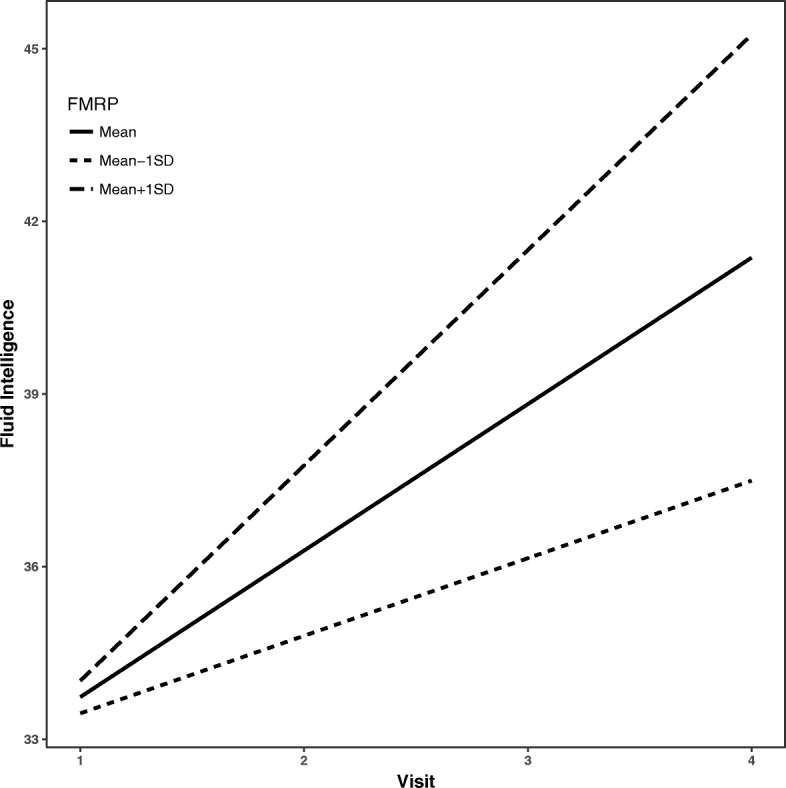
Gains over time in fluid reasoning predicted by level of FMRP expression. Legend: estimated average trajectories over time in fluid intelligence for individuals with FMRP level at the mean (solid), 1 standard deviation (SD) below the mean (short-dashed) and 1 SD above the mean (long-dashed). Higher levels of FMRP were associated with greater improvement in fluid intelligence over time

Fluid intelligence and crystallized intelligence raw scores increased by 2.0 points (*β* = 2.0, SE = 0.7, *p* = 0.02) and 5.2 points (*β* = 5.2, SE = 0.6, *p* < 0.001), respectively, on average per visit. Gains in fluid intelligence raw scores over time were predicted only by biological factors—such that a higher ratio of affected to total chromosomes was associated with a slower rate of improvement in fluid intelligence raw scores (*β* = − 1.5, SE = 0.6, *p* = 0.02) (see Fig. 1) and a higher level of FMRP was associated with a greater increase over time in fluid intelligence raw scores (*β* = 1.2, SE = 0.4, *p* = 0.01) (see Fig. 2). Gains over time in crystallized intelligence raw scores were not predicted by either biological or maternal variables (*p* > 0.05). All data are presented in Table 3.

### Anxiety and withdrawn behaviors at baseline

In terms of genetic predictors, the ratio of affected to total chromosomes, but not FMRP level, was associated with level of withdrawal (*β* = 6.9, SE = 1.7, *p* < 0.001) and anxious/depressed (*β* = 5.5, SE = 1.9, *p* = 0.006) behaviors at baseline. In terms of environmental variables, Child-PAI score (*β* = − 8.1, SE = 1.7, *p* < 0.001) and SCL-90-R GSI T score (*β* = 5.2, SE = 2.2, *p* = 0.02) were each associated with the level of withdrawal behaviors, such that a higher Child-PAI score was associated with a lower CBCL Withdrawn *T* Score, and higher SCL-90-R GSI *T* score was associated with a higher CBCL Withdrawn *T* Score. Finally, there was a trend for higher child-PAI scores to be associated with lower CBCL Anxious/Depressed *T* Score (*β* = − 4.0, SE = 2.0, *p* = 0.053). All data are presented in Table 3.

### Trajectory of anxiety and withdrawal behaviors

CBCL Withdrawn and Anxious/Depressed *T* scores were both stable over time (*β* = − 0.28, SE = 0.75, *p* = 0.72; *β* = − 0.53, SE = 0.67, *p* = 0.44). Thus, there was no need to evaluate predictors of change. All data are presented in Table 3.

## Discussion

The current study aimed to investigate the potential contributions of genetic and maternal-related environmental factors to important dimensions of the cognitive and behavioral phenotype of adolescent females with FXS. For these dimensions, we focused on both the level of functioning related to chronological age expectations and absolute change in skill and problem severity over a 3-year period. At a cross-sectional level, we found that (a) maternal psychological distress, closeness in the mother–child relationship and (b) the active proportion of cells for the X chromosome carrying the full mutation predicted the degree of impairment in fluid intelligence variability at baseline. Interestingly, individual gains over time in fluid reasoning were predicted by the two biological predictors (ratio of affected to total chromosomes and FMRP level), but not by the maternal-related environmental variables. In contrast, degree of impairment at baseline in crystallized intelligence was primarily predicted by the two maternal-related environmental predictors (maternal psychological distress and perceived closeness in the mother–child relationship), but not by the biological variables. Gains over time in crystallized intelligence were not predicted by any of the biological and environmental variables. We also found that withdrawn behavior was stable over time, with the extent of problems in this domain at baseline predicted by the ratio of affected to total chromosomes, perceived closeness in the mother–child relationship, and maternal psychological distress. Anxiety problems also were stable over time, with the extent of the problems predicted by the ratio of affected to total chromosomes.

Our results are generally in line with previous investigations showing that distinct contributions between FMRP and family-related environmental variables contribute differentially to the cognitive and behavioral features of the FXS phenotype [27, 28, 33]. Note that none of these previous studies, however, included the ratio of affected to total chromosomes as a potential predictor, which is preferable to FMRP level, as a metric for females with FXS as an index of biological affectedness [47–49]. Because the ratio of affected to total chromosomes is expressed within a larger range of variability (current sample ranges from 27 to 88 with a SD of 19.9) than the percentage of lymphocytes expressing FMRP (current sample ranges from 0.34 to 0.51 with a SD of 0.05), it is reasonable to hypothesize that the former biomarker should be more sensitive than the latter to the cognitive and/or behavioral variability in the FXS female population. In this regard, some studies have suggested greater cognitive and behavioral difficulties associated with the ratio of affected to total chromosomes in females with FXS [50–53]; however, none of these previous studies distinguished between fluid and crystallized intelligence and whether these domains were differentially affected by this genetic biomarker.

Crystallized intelligence is considered a relative strength compared to a more impaired fluid intelligence in the FXS population [8, 10, 54, 55]. It is also thought that the former domain is more likely to be malleable across the life span through enriched environments than is the latter in the general population [56, 57]. Consistent with this claim we saw that maternal mental health and perception of mother–child closeness were related to crystallized intelligence. Conversely, the only variables that accounted for change over time in fluid intelligence were those of a biological/genetic nature. This unique link is likely to be a consequence of the abnormal frontal lobe development in the FXS population [5, 58].

We hypothesized that the role that enriched environments play in modulating crystallized intelligence may counterbalance the consequences of the biological impairments in females with FXS. In contrast, fluid intelligence requires planning, decision making, problem-solving, abstracting and generalizing rules [59], all of which are cognitive processes that depend on frontal lobe functioning [60], which has been shown to have abnormal structure and function in the FXS population [4, 5, 58, 61, 62].

We also hypothesized that anxiety and social withdrawal would be closely linked to biological factors. In fact, previous studies have found increased anxiety in social situations to be correlated with reduced FMRP [63]. Consistent with these findings, we found that ratio of affected to total chromosomes was significantly associated with both anxiety/depressed and withdrawn behaviors. These biological predispositions do not negate the role or impact of environmental factors. Indeed, we observed a relationship between ratings of withdrawn-depressed behaviors of the adolescent female with FXS and mother’s perception of how close she believed her child felt toward her, as well as maternal symptoms of mental health.

Previous studies have also found associations between maternal mental health status and behavior problems and cognitive development in individuals with FXS [32, 64–66]. It is important to note that these associations likely reflect dynamic bidirectional relationships. It has been well documented that increased levels of psychological stress are associated with parenting a child with a developmental disability [64]. Moreover, there is evidence documenting the negative impact of heightened stress on mental health status, particularly in mothers [34].In addition, mothers of children with FXS are also at increased vulnerability to mental health disorders (i.e., depression, anxiety) due to their own premutation status. In light of these relationships, intervention approaches targeting maternal well-being may help mothers be more resilient in these circumstances, thereby supporting more positive outcomes in children. At the same time, interventions on child behavior may have a positive impact on maternal mental health [67]. Finally, note that although mothers are more likely to be the primary carers of children with ID, current findings are likely to pertain to both parents [68, 69].

In closing, we acknowledge several limitations of this study. First, it is unclear whether blood levels of FMRP are precise indicators of FMRP levels in the brain, in particular as white blood cells are derived from a different embryonic tissue than the brain. In addition, the method for determining FMRP in this study is not strictly quantitative; That is, it reflects the number of cells positive for FMRP rather than the total quantity of FMRP expressed. Second, the SCL-90 is a measure of current maternal psychological distress, a variable that could change significantly over time. It also is possible that scores on the PAI could change over time, reflecting shifts in maternal perception of the parent–child relationship. It would be interesting to track change in maternal stress and psychological state. Indeed, our original intent was to follow up with mothers at the final assessment with these measures, we had a high number of mothers who chose not to participate in subsequent assessments (> 70%); therefore, we decided not to include these Time 4 data in the analyses.

Another limitation of this study is the small sample size. Although replication with a larger sample is needed, limited sample sizes are expected in most studies conducted in special populations such as females with FXS. The small sample size also constrained the approach to the statistical analysis. In particular, we examined the predictors separately rather than together, which would have allowed determining their relative contributions to cognition and behavior. Finally, we examined only a few possible dimensions of the environment and we did not examine more proximal variables, such as the quality of parent–adolescent interactions. Research on this latter area, would have direct implications for intervention.

## Conclusions

To conclude, our results show that FXS biomarkers and maternal variables contribute differentially to the cognitive and behavioral features of the adolescent female with FXS. These findings can help in the design of clinical trials (CTs) aimed at enhancing cognitive and behavioral abilities in the FXS population. For example, the fact that crystallized intelligence is better predicted by maternal environmental factors, whereas fluid intelligence is better predicted by FXS biomarkers, suggests that different interventions may be useful for each. One possibility is that biological interventions may be more directly impactful on fluid intelligence skills, whereas crystallized intelligence skills may require, in addition, environmental enrichment of some sort. However, further investigations with larger samples are needed to confirm our results.

## Abbreviations

ASD: Autism spectrum disorder
CBCL: Child Behavior Checklist
EVT: Expressive vocabulary test
*FMR1*: *Fragile X mental retardation 1* gene
FMRP: Fragile X mental retardation 1 protein
FXS: Fragile X syndrome
GSI: General severity index
IQ: Intellectual quotient
M: Mean
PAI: Positive Affect Index
RP: Repeated Patterns
SCL-90-R: The Symptom Checklist—90 Revised
SD: Standard deviation
SO: Sequential Order
TD: Typically developing
